# A polymorphism in JMJD2C alters the cleavage by caspase-3 and the prognosis of human breast cancer

**DOI:** 10.18632/oncotarget.2029

**Published:** 2014-05-27

**Authors:** Qi Hong, Sanjian Yu, Yu Yang, Guangyu Liu, Zhiming Shao

**Affiliations:** ^1^ Department of Breast Surgery, Fudan University Shanghai Cancer Center; Department of Oncology, Shanghai Medical College, Fudan University, shanghai, China; ^2^ Institutes of Biomedical Sciences, Fudan University, Shanghai, P.R. China; ^3^ School of basic medical sciences, Chengdu University of traditional Chinese medicine, Chengdu, P.R. China

**Keywords:** polymorphism, JMJD2C, caspase-3, cleavage, breast cancer

## Abstract

JMJD2C is a candidate oncogene that encodes a histone lysine demethylase with the ability to demethylate the lysine 9 residue of histone H3 (H3K9). The expression levels of JMJD2C are associated with tumor development and clinical outcome. Here we identify JMJD2C as a new substrate for caspase-3. JMJD2C is cleaved by caspase-3 at DEVD396G motif and then loses its demethylase activity. Additionally, we uncover D396N polymorphism (rs2296067) in the cleavage site of JMJD2C and establish its influence on the resistant to the cleavage by caspase-3. Importantly, we determined that D396N polymorphism is significantly associated with the prognosis of human breast cancer. We further found that the basal levels of DSB (double strand DNA break) repair proteins γ-H2AX (gamma-H2AX) increased when cells were treated with tumor necrosis factor-α (TNF-α) which activates caspase-3 activity. We also show that knockdown of JMJD2C expression results in up-regulation of basal γ-H2AX. We propose that D396N polymorphism of JMJD2C affects the prognosis of human breast cancer via altering the cleavage by caspase-3 and the ability of DSB repair which may contribute to therapy resistance.

## INTRODUCTION

Also known as KDM4C and GASC1, JMJD2C is a member of the Jumonji domain-2 (JMJD2) family and encodes a protein with one JmjC domain, one JmjN domain, two PHD-type zinc fingers and two Tudor domains. JMJD2C has been proved to be a demethylase for H3K9 methylation [1]. It was initially identified as a gene amplified in squamous cell carcinoma and was found to be frequently amplified in several human tumors including breast cancer [2-4]. Although JMJD2C is proved to effectively remove both H3K9me3 and H3K9me2, the H3K9me3 levels do not consist with the relative levels of JMJD2C mRNA in several cell lines [1]. Taking into account that the stability of JMJD2C protein may cause inconsistencies between the mRNA level and protein abundance, the protein modifications of JMJD2C should be studied. The DNA double strand break (DSB) is the principle cytotoxic lesion for ionizing radiation or many cancer chemotherapeutic agents which induce DNA damage [5, 6]. The most important process for the survival of cancer cells following DNA damage is the DSB repair. The resistance to radiotherapy and chemotherapy for cancer cells is often mediated by enhanced DSB repair [7-9]. On DNA damage, H3K9me3 is involved in DSB repair by binding Tip60 acetyltransferase, which activates ATM (ataxia telangiectasia mutated) kinase and initiates a signaling cascade that regulates DSB repair [10, 11].

Caspase-3 is a primary effector of the cysteine protease family, which plays an essential role during apoptotic cell death by proteolytic cleaving a variety of key proteins required for cellular functioning and survival [12]. For example, caspase-3 cleaves nuclear enzyme poly (ADP-ribose) polymerase 1 (PARP-1) by recognizing the DEVDG motif within DNA-binding domain of PARP-1 and separating the two fragments to inactivate the enzymatic activity of poly(ADP-ribosyl)ation [13, 14]. GATA-1, a member of GATA family of transcription factors, plays an important role in erythroid development. It is also be cleaved by caspase-3 to regulate differentiation of erythroid cells [15]. Although apoptosis has been suggested to be the barrier to tumor initiation, progression and metastasis, recent studies also demonstrated unexpected functions of caspase-3 in tumors [16]. Caspase-3 was found to stimulate tumor repopulation during cancer radiotherapy [17]. The levels of caspase-3 were found significantly higher in carcinomas compared with fibroadenomas and adjacent normal breast tissues [18]. We suppose that cleavage of many key proteins by caspase-3 might be involved in cancer tumorigenesis or progression. Thus, the identification of new cleavage substrates for caspase-3 would lead to further insights into the regulation and function of proteins in cancer.

In this report, we identify and describe a SNP rs2296067 (D396N) in caspase-3 cleavage site of JMJD2C that affects its cleavage by caspase-3. The cleavage of JMJD2C by caspase-3 increases the levels of H3K9me3 and basal levels of γ-H2AX (an early event that is mediated by ATM in DSB-induced signaling cascade). In addition, we observed that D396N polymorphism of JMJD2C is significantly associated with the prognosis of human breast cancer. This study demonstrates that D396N polymorphism makes JMJD2C resistant to caspase-3 cleavage, thereby revealing a functional connection between prognosis of breast cancer, caspase cleavage and DSB repair.

## RESULTS

### JMJD2C is cleaved by caspase-3-like protease

**Figure 1:**
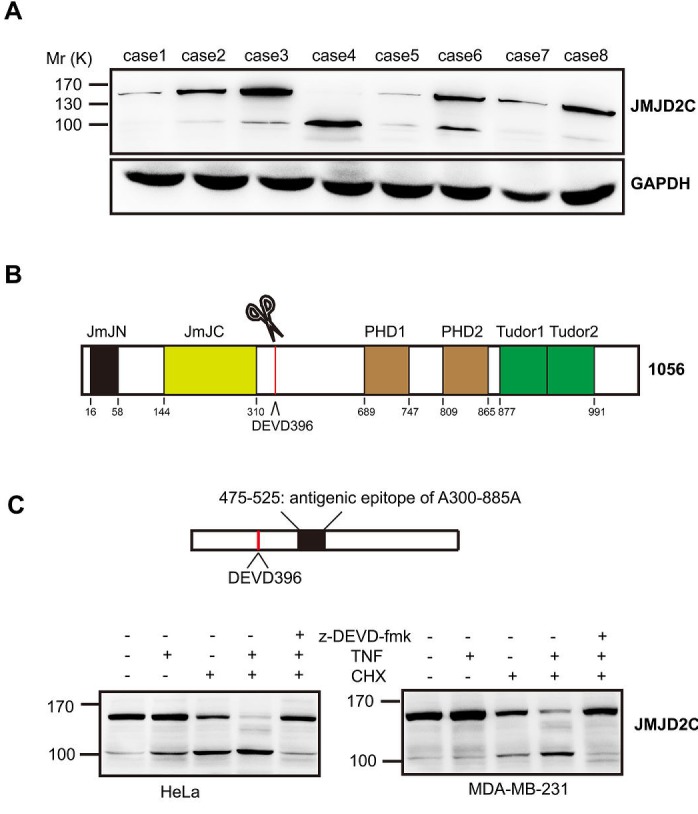
JMJD2C is cleaved by caspase-3-like protease (A) Expression of JMJD2C protein in 8 breast-tumor samples was analyzed by western blotting with anti-JMJD2C (A300-885A) antibody. (B) Schematic representation of the structure of JMJD2C. (C) Schematic representation of A300-885A antibody mapping. Cell lysates of HeLa and MDA-MB-231 were analyzed by western blotting with A300-885A antibody. Where indicated, cells were treated with 20 mM z-DEVD-fmk, for 2 h before treatment. HeLa and MDA-MB-231 cells were treated with TNF-α (25 ng/ml) or CHX (10 μg/ml) for 6 h or with the combination for 6 h.

For the purpose to examine the protein expression of JMJD2C in breast-tumor samples, we first performed western blotting analysis to test the JMJD2C antibody (A300-885A). Interestingly, two specific fragments were found in several samples (Fig. 1A). High levels of large fragments with relative molecular mass ~150,000 (*M*_r_ ~150K) were present in most samples. Meanwhile, the small fragments with relative molecular mass ~100,000 (*M*_r_ ~100K) were detected at low levels in most samples. Only the small fragment of JMJD2C was detected in the case 4 sample. By analyzing the amino acid sequence of JMJD2C, a caspase-3-like protease cleavage sequence DEVD396G (Asp-Glu-Val-Asp-Gly) was found to be located downstream of the JmJC domain of JMJD2C (Fig. 1B). The epitope of A300-885A antibody is located downstream of DEVD396G motif (between residue 475 and 525, Fig. 1C). A300-885A antibody seems to recognize both of the native and the C-terminal cleavage fragments of JMJD2C. Thus, we suppose that the two specific fragments detected by A300-885A antibody were generated by the caspase-3-like protease cleavage of JMJD2C.

To test the hypothesis that JMJD2C is cleaved by caspase-3-like protease, we first carried out western blotting analysis on total cell lysates of HeLa cell lines or MDA-MB-231 breast cancer cell lines that were stimulated with tumor necrosis factor-α (TNF-α)/cycloheximide (CHX) to induce caspase-3-like protease activity. The *M*_r_ 100K fragment of JMJD2C was detected using the A300-885A antibody treated with TNF-α or CHX alone for 6 h (weakly in MDA-MB-231 cells treated with TNF-α alone) (Fig. 1C). Meanwhile, apparent increase of *M*_r_ 100K fragment of JMJD2C was observed after treatment with TNF-α plus CHX (TNF-α/CHX) for 6 h, whereas the abundance of the native *M*_r_ 150K fragment decreased markedly (Fig. 1C). In addition, cleavage of JMJD2C was found to be dependent on caspase-3-like-dependent proteolysis, as the generation of *M*_r_ 100K fragment was inhibited in the presence of z-DEVD-fmk (caspase-3 inhibitors) (Fig. 1C). These results indicate that caspase-3-like protease seems to be responsible for the cleavage of JMJD2C.

### The JMJD2C D396N polymorphism located at the potential caspase-3-like protease cleavage site

**Figure 2:**
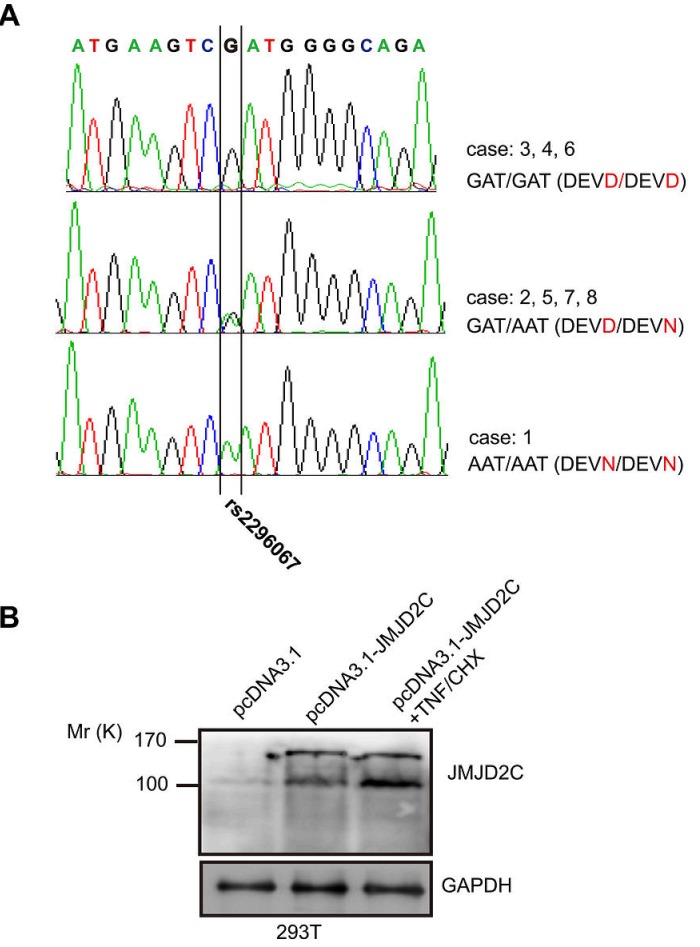
The JMJD2C D396N polymorphism in potential caspase-3-like protease cleavage site (A) SNP rs2296067 in 8 breast-tumor samples (B) 293T cells were transiently transfected with empty pcDNA3.1 vector and pcDNA3.1-JMJD2C carrying G allele of the SNP rs2296067. After the transfection for 24 hours, the pcDNA3.1-JMJD2C transfected cells were treated with TNF-α (25 ng/ml) plus CHX (10 μg/ml) for 2 h. Cell lysates were analyzed by western blotting with anti-JMJD2C (A300-885A) antibody.

We isolated the full length of JMJD2C cDNA to do overexpression analysis with the cDNA from case 2 sample. After the sequencing, we got two kinds of clones with nucleotide bases A or G in the same site of JMJD2C cDNA (1188 site). The two different nucleotide bases (G/A) result in amino acid changes in the cleavage motif (DEVD/N396G) of JMJD2C. By searching the NCBI dbSNP database for polymorphisms in the sequences of JMJD2C gene, we found that a single nucleotide polymorphism rs2296067 (D396N) was responsible for this. By sequencing the exon-9 of JMJD2C gene that contains DEVD396 motif in the above samples, three types of genotype of SNP rs2296067 were shown in the above breast-tumor samples (Fig. 2A). Furthermore, HeLa and MDA-MB-231 cells were found to carry homozygous G/G SNP rs2296067 (data not shown).

We then carried out western blotting analysis on total cell lysates of 293T cells that were transiently transfected with D396 variant of JMJD2C. *M*_r_ 150K fragment and *M*_r_ 100K fragment were detected increased by using A300-885A antibody after the transfection (Fig. 2B). With the treatment of TNF-α/CHX, *M*_r_ 100K fragment was found increased after the stimulation for 2 h (Fig. 2B). These findings support the hypothesis that JMJD2C is cleaved by caspase-3-like protease. Based on our hypothesis, the protein product of JMJD2C gene with “A” allele of SNP rs2296067 may be resistant to the cleavage by caspase-3-like protease.

### JMJD2C is cleaved by caspase-3 at DEVD396G motif

**Figure 3:**
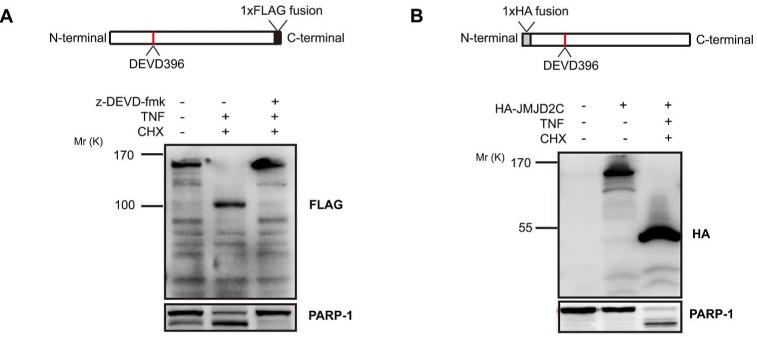
Caspase-3-like protease cleavage removes a C-terminal fragment from JMJD2C (A) HeLa cells were stably transfected with full length JMJD2C (D396) tagged with FLAG at the C-terminus. The top schematic representation shows the C-terminal FLAG epitope tag of JMJD2C. Where indicated, HeLa cells were treated with 20 mM z-DEVD-fmk for 2 h before treatment and then were treated with TNF-α (25 ng/ml) plus CHX (10 μg/ml) for 6 h. Cell lysates of HeLa were analyzed by western blotting with FLAG antibody. Endogenous levels of total full-length PARP-1 and the large fragment produced by caspase-3 family protease cleavage at Asp214 were detected by PARP antibody. (B) HeLa cells were stably transfected with full length JMJD2C (D396) tagged with HA at the N-terminus. The top schematic representation shows the N-terminal HA epitope tag of JMJD2C. Treated with TNF-α (25 ng/ml) plus CHX (10 μg/ml) for 6 h, cell lysates of HeLa were analyzed by western blotting with HA antibody. The untransfected and untreated cells were set as the control. Full-length and the cleavage fragment of PARP-1 were also detected.

The putative DEVD396G cleavage motif, together with the inhibitory effect of z-DEVD-fmk, indicates that JMJD2C may be cleaved at the DEVD396G motif by a caspase-3-like protease. We stably transfected HeLa cells with D396 variant of JMJD2C tagged with FLAG at the C-terminus. With the treatment of TNF-α/CHX, FLAG antibodies revealed the native (*M*_r_ ~150K) and the C-terminal cleavage fragments (*M*_r_ ~100K) of JMJD2C. In the presence of z-DEVD-fmk, the generation of C-terminal cleavage was inhibited. Meanwhile, PARP-1, a well-characterized caspase-3 substrate, was cleaved in a similar manner to JMJD2C (Fig. 3A). These results are consistent with the findings using A300-885A antibody to detect endogenous JMJD2C. Furthermore, we stably transfected HeLa cells with D396 variant of JMJD2C tagged with HA at the N-terminus. Transfected cells were treated with TNF-α/CHX, and then the total proteins were subjected to western blotting analysis with HA antibodies. A fragment of *M*_r_ ~50K was observed as well as the *M*_r_ ~150K native fragment was not detected in the presence of TNF-α/CHX (Fig. 3B). Thus, the cleavage of JMJD2C by caspase-3-like protease was further confirmed.

**Figure 4:**
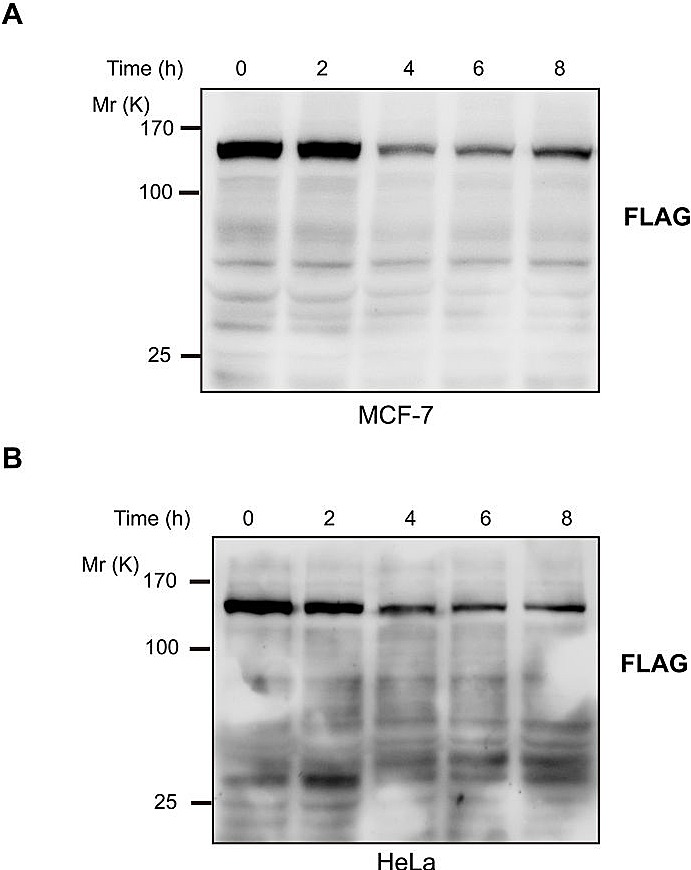
JMJD2C is cleaved at DEVD396G motif by caspase-3 (A) MCF-7 cells were stably transfected with full length JMJD2C (D396) with C-terminal FLAG epitope tag. Treated with TNF-α (25 ng/ml) plus CHX (10 μg/ml) for 2, 4, 6 and 8 h, the cell lysates of MCF-7 were analyzed by western blotting with FLAG antibody. (B) HeLa cells were stably transfected with full length JMJD2C (N396) with C-terminal FLAG epitope tag. The transfected HeLa cells were treated with TNF-α (25 ng/ml) plus CHX (10 μg/ml) for 2, 4, 6 and 8 h. Cell lysates were analyzed by western blotting with FLAG antibody.

As the result of a deletion in exon 3 of the caspase-3 gene, MCF-7 human breast cancer cell line lacks caspase-3 activity [19]. We stably transfected MCF-7 cells with D396 variant of JMJD2C tagged with FLAG at the C-terminus. Treatment with TNF-α/CHX of these cells, we could not detect the cleavage of JMJD2C (Fig. 4A). Thus, caspase-3 seems to be responsible for the cleavage of JMJD2C. To determine whether JMJD2C is cleaved by caspase-3 in the DEVD396G motif, we introduced a point mutation by substituting an asparagine for aspartate at position 396 in the JMJD2C protein (D396N). We generated stable HeLa cell lines expressing mutated JMJD2C (N396) tagged with FLAG at the C-terminus. As shown in Fig. 4B, we could not detect the cleavage fragments from 2 to 8 h after TNF-α/CHX stimulation using FLAG antibodies. These results indicate that point mutation by substituting an asparagine for aspartate at position 396 of JMJD2C prevents the cleavage of JMJD2C by caspase-3.

### Cleavage of JMJD2C by caspase-3 inactivates its demethylase activity

**Figure 5:**
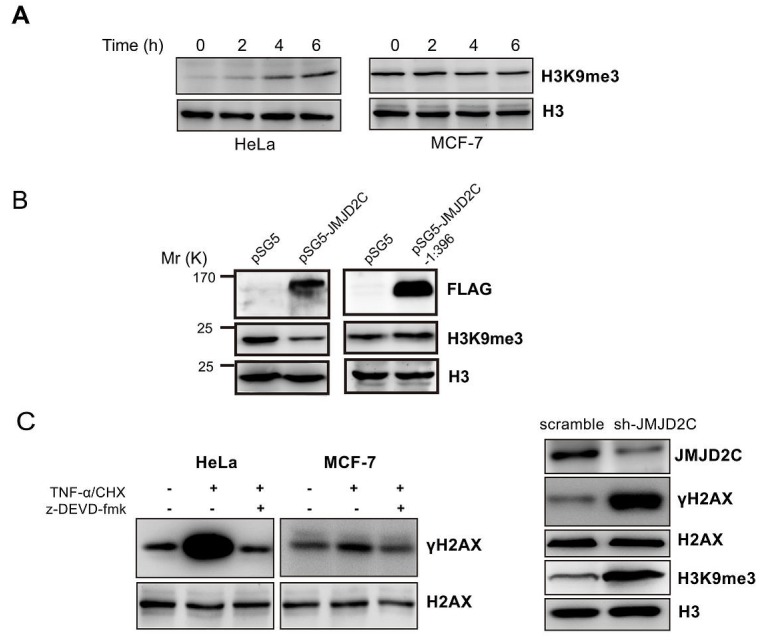
Cleavage of JMJD2C by caspase-3 inactivates its demethylase activity (A) HeLa and MCF-7 cells were treated with TNF-α (25 ng/ml) plus CHX (10 μg/ml) for 2, 4 and 6 h. The treated cells were lysed and then were subjected to western blotting analysis with H3K9me3 antibody and histone H3 antibody. Levels of histone H3 were taken as loading control. (B) MCF-7 cells were transiently transfected with the empty vector or full-length JMJD2C with N-terminal FLAG epitope tag or N-terminal cleavage JMJD2C (fragment 1-396 amino acid) with N-terminal FLAG epitope tag. The expression of JMJD2C (full length and fragment 1-396 amino acid), H3K9me3 and histone H3 were analyzed by western blotting with corresponding antibodies as indicated. Levels of histone H3 were taken as loading control. (C) Western blotting analysis demonstrated that the levels of γ-H2AX was significantly elevated during the treatment with TNF-α/CHX in HeLa cells but not in MCF-7 cells (left panel); HeLa cells were stably transfected with shRNA control (scramble) or with shRNA targeting JMJD2C (shJMJD2C). Western blotting analysis showing expression JMJD2C, H3K9me3, H2AX, γ-H2AX and histone H3 were analyzed by western blotting with corresponding antibodies as indicated. Levels of histone H3 were taken as loading control (right panel).

In the present study we have shown that JMJD2C is cleaved by caspase-3 in DEVD396G motif. Therefore, we test whether the cleavage of JMJD2C possesses lysine demethylase activity on histone H3. TNF-α/CHX treated cell lysates were subjected to western blotting analysis and the levels of histone H3 were taken as the loading control. A marked increase of the levels of H3K9me3 was detected in TNF-α/CHX treated HeLa cells. Differently, the levels of H3K9me3 was not changed in TNF-α/CHX treated MCF-7 cells (homozygous G/G SNP rs2296067, data not shown) (Fig. 5A). These findings imply that cleavage of JMJD2C by caspase-3 may eliminate its demethylase activity to H3K9. A previous study has shown that the truncation (fragment 2-660 amino acid) of JMJD2C that lacks both Tudor and PHD domains retains histone demethylase activity [20]. Thus, MCF-7 cells were transiently transfected with full-length JMJD2C or N-terminal cleavage JMJD2C (fragment 1-396 amino acid). As shown in Fig. 5B, overexpression of full-length JMJD2C but not N-terminal cleavage JMJD2C could significantly reduce the levels of H3K9me3. Thus, cleavage of JMJD2C by caspase-3 seems to inactivate its demethylase activity.

H3K9me3 is involved in DSB repair by binding Tip60 acetyltransferase, which activates ATM and initiates a signaling cascade that regulates DSB repair [10, 11]. The generation of γ-H2AX is the early event that is mediated by ATM in DSB-induced signaling cascade [21]. With the treatment of TNF-α/CHX, we found a significant increase of the basal levels of γ-H2AX in HeLa cells. This effect could be inhibited by caspase-3 inhibitor z-DEVD-fmk. Nevertheless, the basal levels of γ-H2AX did not change in caspase-3-deficient MCF7 cells after treatment with TNF-α/CHX (Fig. 5C). We further showed that knockdown of JMJD2C resulted in significantly higher levels of H3K9me3 and γ-H2AX in HeLa cells (Fig. 5C). Thus, the cleavage of JMJD2C may inactivate the demethylase activity and increase H3K9 methylation levels and hence the levels of γ-H2AX.

### Association of SNP rs2296067 with breast cancer patient survival

**Figure 6:**
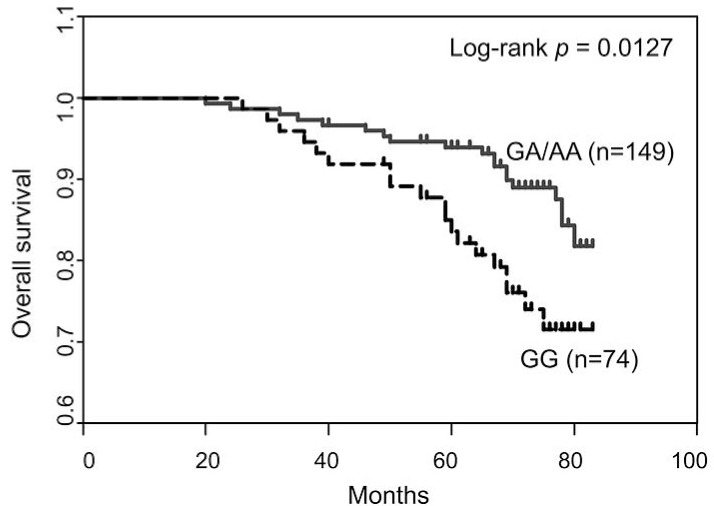
Association of SNP rs2296067 with breast cancer patient survival Kaplan–Meier analysis of the overall survival of breast cancer patients grouped according to their genotype for SNP rs2296067.

Next we examined the distribution of SNP rs2296067 in breast cancer patients (n=223) and healthy controls (n=162). SNP rs2296067 was found at relatively high frequency in the heterozygous state (G/A, 48.1%) and homozygous state (G/G, 32.1%) in the healthy controls (Table 1). Additionally, the frequency of SNP rs2296067 in breast cancer patients was found to be similar to the frequencies found in the healthy controls (Table 1). Thus, we further analyzed the prognosis of these breast cancer patients. In the Kaplan-Meier analyses, SNP rs2296067 was significantly associated with OS. The patients with homozygous G/G genotype exhibited a decreased postoperative OS compared with those with non-homozygous G/G genotype (Fig. 6). This indicates that the cleavable form of JMJD2C may attenuate therapeutic efficacy in breast cancer patients.

**Table 1:** Genotype frequencies of rs2296067 in breast cancer and healthy controls

Genotypes controls	Breast cancer	Healthy
GG	74 (33.2%)	52 (32.1%)
GA	110 (49.3%)	78 (48.1%)
AA	39 (17.5%)	32(19.8%)

## DISCUSSION

In the present study, we have shown that JMJD2C is cleaved by caspase-3 at DEVD396G motif. Cleavage of JMJD2C inactivates the H3K9 demethylase activity and elevates the levels H3K9me3. Moreover, the SNP rs2296067 (D396N) which results in the aspartate to asparagine acid substitution at position 396 of JMJD2C protein was found in JMJD2C gene. This SNP creates a substitution in the caspase-3 cleavage motif of JMJD2C (DEVD/N396G). Previous studies have shown that aspartate to asparagine acid mutation at position 214 (D/N214) in DEVD214G motif of PARP-1 renders it resistant to the cleavage by caspase-3. Similarly, the JMJD2C variant carrying DEVN396G motif is also resistant to the cleavage by caspase-3.

JMJD2C was identified as a gene frequently amplified in different types of cancer such as oesophageal squamous cell carcinoma (ESCC) [2], acute myeloid leukemia (AML) [22], primary mediastinal B cell lymphoma (PMBL), Hodgkin lymphoma (HL) [23] and breast cancer [3, 4]. Although JMJD2C was proved to demethylate H3K9 methylation, the expression of JMJD2C mRNA does not consist with the global levels of H3K9me3 in several cell lines [1]. This study suggests that cleavage of JMJD2C by caspase-3 may be the reason for the inconsistency between the levels of JMJD2C mRNA and H3K9me3.

Interestingly, previous studies have shown that caspase-3 activity is critical to differentiation of ESCs (Embryonic stem cells) and the differentiation may depend on caspase-3-induced cleavage of Nanog in differentiating ESCs [24]. H3K9 methylation is essential for establishment and maintenance of the silent chromatin state and H3K9me3/2 is maintained at a low level in ESCs. During the differentiation of ESCs, a sustained increase of H3K9me3/2 is observed. Furthermore, Depletion of JMJD2C was found to promote the differentiation of ESCs [25]. These findings imply that cleavage of JMJD2C by caspase-3 may increase the levels of H3K9 methylation and hence promote the transition of the transcriptionally active chromatin into silent chromatin states during the differentiation of ESCs.

In many, but not all JMJD proteins, the JmjC domain has the catalytic activity and mediates the demethylation of histone lysine residues [26]. A previous study has shown that the truncation (fragment 2-660 amino acid) of JMJD2C with the deletion of Tudor and PHD domains retains the histone demethylase activity [20]. We transfected MCF-7 cells with the N-terminal cleavage JMJD2C (fragment 1-396 amino acid). As shown in Fig. 5B, overexpression of N-terminal cleavage JMJD2C did not demethylate the tri-methylation of lysine 9 on histone H3. This observation suggests that cleavage of JMJD2C by caspase-3 inactivates its demethylase activity to H3K9 methylation.

In this study, the JMJD2C SNP rs2296067 was found in the cleavage site of JMJD2C. The protein product of JMJD2C with the minor “A” allele in SNP rs2296067 is resistant to the cleavage by caspase-3. Accordingly, it seems like that the individuals carrying homozygous G/G genotype in SNP rs2296067 may suffer prolonged lysine 9 demethylation on histone H3. Thus, levels of H3K9me3 may be reduced in the individuals carrying homozygous G/G genotype in SNP rs2296067. Chemotherapeutic drugs and radiation cause DNA damage and induce signaling pathways for apoptotic cell death. Enhanced DNA damage repair processes play a role in the development of resistance to chemotherapy and radiation therapy [8]. H3K9me3 is involved in the initial processing of DNA repair [11]. During the treatment with TNF-α/CHX, the protein product of JMJD2C with the minor “G” allele in SNP rs2296067 undergoes cleavage by caspase-3. Meanwhile, the basal levels of γ-H2AX also increases. Furthermore, we found that the patients with homozygous G/G genotype in SNP rs2296067 of JMJD2C had worse OS compared with those with non-homozygous G/G genotype. These imply that the cleavage JMJD2C leads to accumulated levels of H3K9 methylation and enhanced ability of repair of DNA DSB. Thus the patients carrying only cleavable JMJD2C are resistant to chemotherapy and radiation therapy.

Our findings reveal a new mechanism for negative regulation of JMJD2C by caspase-3 cleavage. Moreover, the single nucleotide polymorphism rs2296067 of JMJD2C renders it resistant to the cleavage by caspase-3. Taken together, these results may lead to further insights into the regulation and function of JMJD2C in cancer and stem cells.

## MATERIALS AND METHODS

### Cell lines and reagents

The human breast cancer MCF-7 and MDA-MB-231 cell lines, 293T and HeLa cell lines were obtained from the American Type Culture Collection. MDA-MB-231 cell lines were maintained in continuous culture in L-15 medium supplemented with 10% fetal bovine serum (FBS). Other cells were maintained in continuous culture in MDEM medium supplemented with 10% FBS. These cell cultures were maintained in a 37°C incubator with a humidified 5% CO_2_ atmosphere. Rabbit anti-JMJD2C (A300-885A) was from Bethyl Laboratories. Mouse anti-H3, anti-H3K9me3, anti-GAPDH, anti-FLAG and anti-HA were from Abmart Company. Rabbit anti-PARP-1 was obtained from Cell Signaling Technology. z-DEVD-fmk was from BD Bioscience. Cycloheximide was from Amresco. Recombinant Human TNF-α was from PeproTech. Polybrene and puromycin were from Sigma.

### DNA preparation and genotyping

Genomic DNA from 223 patient tumor samples with known clinical history was extracted manually using the QIAamp DNA Mini Kit (Qiagen) according to the manufacturer's instructions. Genomic DNA from the blood leukocytes of 162 participants (healthy controls) was extracted Gentra's PureGene DNA Purification Kit (Gentra systems). Genotypes of SNP rs2296067 were determined by direct sequencing of exon-9 of JMJD2C gene from PCR-amplified genomic templates. Primer sequences used for PCR- amplification were 5'-TTCATATCCATTGCAGACATCCCGC-3' (also for the sequencing); 5'-AGGCACTGCTGACTGCTTGTCT-3'. This study was approved in advance by the Institutional Review Board of the Cancer Hospital, Fudan University.

### RNA isolation and vector construction

Extraction and reverse transcription of the Total RNA were done with TRIzol reagent (Invitrogen) and PrimeScript RT reagent Kit (Takara Biotechnology). The pSG5 (2xFLAG epitope tag in N-terminus) plasmid is a gift from Jiemin Wong (East China Normal University). The human full-length cDNA of JMJD2C carrying “G” allele in SNP rs2296067 was obtained from the cDNA in case 2 breast-tumor sample and then cloned into the vectors. In-Fusion HD cloning kit (Takara) was used for homologous recombination between amplified fragments and the vectors. The primers were: pcDNA3.1 (5'-GCCCTCTAGACTCGAATGGAGGTG GCCGAGG-3'; 5'- CAGTGTGGTGGAATTTCACTGTCTCTTCTG-3'), pSG5 (5'- TACCTCTAGAGAATTCAGCGCCGACACCGA GGTGGCCGAGGTGGAAA-3'; 5'-CGGGCGGCCGCTCGAGCTACTGTCT CTTCTGGCACTT-3'). To overexpress the N-terminal cleavage JMJD2C in pSG5, the reverse primer was used: 5'- CGGGCGGCCGCTCGAGCTAATCG ACTTCATCTGACACTT-3'. The cDNA of JMJD2C were subcloned into lentiviral expression plasmid pCDH-CMV (from System Biosciences and adding n-1xHA or c-1xFLAG by our lab) to generate JMJD2C with C-terminal FLAG epitope tag and N-terminal HA epitope tag. The primers were: pCDH-CMV-c-FLAG (5'- TAGCCCGGGCGGATCATGGAGGTGG CCGAGGTGGAA −3'; 5'-AATCTCTAGACTCGACCTGTCT CTTCTGGCACTTCTT-3'), pCDH-CMV-n-HA (5'-TAGCCCGGGCGGATCCGAGGTGGC CGAGGTG-3'; 5'-CGACGATATCGAATTCTACTGTCTCT TCTGGCACTT-3'). Lentiviral vectors (pLKO.1) containing shRNA sequences targeting JMJD2C (shJMJD2C, target sequence: CCTTGCATACATGGAGTCTAA; hairpin sequence: 5'-CCGGCCTTGCATACATGGAGTCTAAC TCGAGTTAGACTCCATGTATGCAAGGTTTTT-3') or non-silencing control scramble shRNA (scramble; hairpin sequence: 5'-CCTAAGGTTAAGTCGCCCTCGC TCGAGCGAGGGCGACTTAACCTTAGG-3') were obtained from Open Biosystems. JMJD2C D396 was mutated to N396 using the primer 5'-GTCAGATGAAGTCAATGGGGCAGAGGTC-3' and the complementary primer 5'-GACCTCTGCCCCATTGACTTCATCTGAC-3' with the Site-directed Gene Mutagenesis Kit (Genesent) and the substitution was confirmed by sequencing.

### Transfection and lentivirus transduction

Transfections were done using Lipofectamine 2000 transfection reagents (Invitrogen) according to manufacturer instructions. The lentiviral expression vector was packed into HEK 293T cells to generate corresponding lentiviruses. The lentiviruses were added to cells with 8 μg/ml polybrene. 48 h later, the cells were changed to fresh puromycin-containing media (2.5μg/ml). After puromycin selection for 72 h, the lentivirus-free cells have completely died. The survived cells added with lentivirus were identified by western blotting analysis for the expression of JMJD2C.

### Western blotting analysis

Cells were lysed for 20 min on ice in lysis buffer (Genesent) with 0.5 mM phenylmethylsulfonyl fluoride (PMSF), 1 mM dithiothreitol, 10 mM NaF, 2m M Na_3_VO_4_, and 5 mg/ml leupeptin. Then cell lysates were centrifuged at 14000×g for 10 min at 4 °C. Protein concentrations were estimated using protein-dye (Bio-Rad). Samples were separated by SDS-PAGE and then blotted onto a PVDF membrane (Millipore). After blotted with primary antibodies overnight, peroxidase conjugated secondary antibodies were incubated with the membrane. Bound antibodies were detected by Chemiluminescent HRP Substrate (Millipore) and photographed by LAS-3000 luminescent image system (Fujifilm).

### Statistical analysis

ANOVA and Student t tests were used to determine the statistical significance of differences between experimental groups. The Kaplan-Meier method was used to analyze breast cancer patient cumulative survival rate. A probability value of 0.05 or less was considered significant. Statistical analysis and graphs were created with GraphPad Prism (Graph-Pad Software).
